# Challenges and coping strategies among young adults living with perinatally acquired HIV infection in Botswana. A qualitative study

**DOI:** 10.1371/journal.pone.0284467

**Published:** 2023-04-26

**Authors:** Grace Karugaba, Gloria Thupayagale-Tshweneagae, Mary M. Moleki, Mogomotsi Matshaba

**Affiliations:** 1 Botswana Baylor Children’s Clinical Centre of Excellence, Gaborone, Botswana; 2 Department of Health Studies, University of SouthAfrica, Pretoria, South Africa; 3 Baylor College of Medicine, Paediatric Retrovirology, Houston, Texas, United States of America; Torrens University Australia, AUSTRALIA

## Abstract

**Background:**

Due to antiretroviral therapy, many people with perinatally acquired HIV are surviving into young adulthood which is a critical period of human development. Research conducted in various settings globally has shown that young adults living with perinatally acquired HIV (YALPH) face multiple challenges related to HIV infection while also confronting the same challenges of young adulthood faced by other HIV-negative youth. However, there is a paucity of information on YALPH in Botswana and what needs to be done to improve their health and wellbeing. Therefore, this study explores the challenges and coping strategies of YALPH in order to inform health policies and programming in Botswana.

**Methods:**

In-depth interviews were conducted with 45 YALPH (ages 18–27 years) who were enrolled on antiretroviral therapy at the Botswana-Baylor Children’s Clinical Centre of Excellence (Botswana-Baylor Clinic). The Botswana-Baylor Clinic is the largest centre for pediatric, adolescent, and young adult HIV treatment and care in Botswana. The maximum variation sampling method was used to select information-rich participants. The questions focused on the challenges YALPH faced and how they coped with HIV. The data was analyzed using content analysis.

**Results:**

The results showed that the majority of YALPH had suppressed HIV viral load and perceived themselves to be in good physical health and functioning. They did, however, face numerous challenges, including occasional or longstanding poor antiretroviral therapy adherence, disabilities and impairments, poor school performance and attainment, unemployment, financial stressors, fear of stigma, disclosure worries and concerns, and limited social support. The most vulnerable YALPH included those with disabilities and impairments, those transitioning out of residential care, young parents, the unemployed, and those with maladaptive coping strategies. The YALPH mainly used adaptive coping strategies. The most commonly used maladaptive coping strategies were self-distraction and venting.

**Conclusion:**

Interventions to prevent, screen for, assess, and manage the challenges identified by this study are critical to improving the health and well-being of YALPH. In addition, diverse interventions that can contribute to the development of adaptive coping mechanisms and reduce the likelihood of maladaptive coping in YALPH should be sought.

## Introduction

Due to the successful antiretroviral therapy (ART) program in Botswana, a large number of perinatally HIV-infected individuals are entering young adulthood. Young adults living with perinatally acquired HIV (YALPH) face complex issues related to coping with a chronic, stigmatizing, and transmittable infection while navigating the developmental tasks of this stage of life. Young adulthood (spanning the ages of approximately 18–30 years) is a critical period of human development, with long-lasting implications for a person’s financial security, health, and well-being [1,2]. During young adulthood, women and men are normally expected to finish school, leave their family homes, find employment, build a network of relationships, start families, and assume other responsibilities that are typical of adult life [1,2]. In Botswana, the beginning of young adulthood coincides with the age at which individuals are legally considered adults [3]. At this age, youth exit the Government of Botswana’s Orphans and Vulnerable Children Support Program and either transition to the Destitute Persons Program, live independently, or find other sources of support. Living with a chronic illness such as HIV during this period can interfere with the achievement of these developmental milestones, creating stress for individuals and marginalization by society [1,2,4].

Research in many settings globally has shown that YALPH face a number of challenges including long-term adherence to ART and viral suppression [5–7], HIV stigma and discrimination [8–10], mental health issues [6,11], neurocognitive deficits [12–14], preventing HIV transmission to sexual partners and offspring [9,15], physical disabilities and impairments [16], and settling down after transitioning from pediatric and adolescent care to adult care settings [5,11]. In addition to HIV-related challenges, YALPH face similar challenges to their uninfected peers such as unemployment, financial challenges, food insecurity, alcohol and substance use, limited education and training opportunities, limited access to recreational and sports opportunities, and poor access to business opportunities [17,18]. Furthermore, it has been noted that young adults have higher levels of daily stressors than older adults and perceive their lives to be more stressful [19,20]. Taken together, these examples suggest that YALPH face significant burdens, emphasizing the importance of research to inform targeted support for this population.

Limited information exists regarding HIV prevalence among young adults in Botswana due to the lack of national-level surveillance data focusing specifically on this population. In general, Botswana has one of the highest HIV prevalence rates in the world. According to the most recent data from the Joint United Nations Program on HIV/AIDS (UNAIDS), Botswana had an estimated 360,000 people living with HIV (PLWH) in 2021, and the HIV prevalence among adults (15–49 years) was 18.6% [21]. In response to the high disease burden, Botswana has made tremendous progress in providing treatment and care services for PLWH. The Botswana National Anti-Retroviral Therapy program was established in 2002 with the goal of reducing HIV-related morbidity and mortality and improving the well-being of PLWH. The program provides universal free ART to PLWH under the "Treat All" policy. This is in addition to the various psychosocial support programs provided by the Government of Botswana and other partners to improve the health and well-being of PLWH. Furthermore, the Government of Botswana has established a number of youth development policies and programs (education, employment, and entrepreneurship) with a special dispensation for vulnerable youth, including HIV-positive youth [17].

In 2021, the World Health Organization (WHO) awarded Botswana the "Silver Tier" status for lowering the mother-to-child transmission of HIV (MTCT) rate to less than 5% and providing prenatal care and ART to more than 90% of pregnant women living with HIV, which moves it closer to the elimination of MTCT [22]. Furthermore, preliminary results from the fifth Botswana AIDS Impact Survey (BAIS V, 2021) show that among adults (ages 15–64 years) living with HIV in Botswana, 95% were aware of their status, 98% of those aware of their status were on ART, and 97.9% of those on ART achieved viral load (VL) suppression [23]. Based on these findings, Botswana has surpassed the UNAIDS 95-95-95 ART and virological suppression targets for 2025 and is on track to achieving epidemic control. However, despite those significant achievements in Botswana’s ART program in reducing morbidity and mortality among PLWH, little attention has been paid to measuring and monitoring the qualitative outcome of ART and other interventions on YALPH well-being, possibly due to the fact that this is still an emerging population. As a result, it is unknown what challenges the YALPH in Botswana face, how they cope with HIV, and what interventions are needed to improve their health and well-being.

Coping is defined as constantly changing cognitive and behavioral efforts to manage specific external and/or internal demands that are taxing or exceeding the resources of a person [20]. Coping may significantly increase or decrease the effects of stress or adverse events, as different types of coping strategies can have protective or harmful effects on individuals’ health and well-being [19,24,25]. Essentially, coping can be classified as either adaptive or maladaptive [26,27]. Coping is adaptive when it serves protective functions and resolves or reduces the negative effects of a stressor, and it is maladaptive when it exacerbates the effects of stress and thus represents additional risk factors [26–28]. For PLWH, adaptive coping strategies have been associated with adaptation to the illness, medication adherence, better mental health, and a higher quality of life [28,29]. On the other hand, maladaptive coping strategies are known to negatively impact mental health, lead to social isolation, and worsen disease symptoms [19,28]. Previous research conducted in other settings globally has shown that coping strategies adopted by HIV-positive youth have important practical applications and are central to HIV management [30–34]. However, no research has yet examined the coping strategies of YALPH in Botswana.

Therefore, the primary purpose of this study was to draw attention to a growing cohort of YALPH that is not well represented in research among PLWH in Botswana. The study provides in-depth information about the challenges and coping strategies of YALPH in Botswana. The perspectives of YALPH on how to address the challenges they faced and how their wellbeing could be improved are also provided. A precursor to improving the wellbeing of YALPH is knowledge of the factors that influence it, upon which appropriate interventions could be developed. The findings of this study can be used by the Ministry of Health (MOH) and other providers to guide the needs-based allocation of resources and the design of policies, programs, and interventions aimed at improving the well-being of YALPH, thereby incorporating the voice of the service recipients into decision-making.

## Methods

### Conceptual framework

The Ferrans Conceptual Model of Health-Related Quality of Life (HRQOL) [35] was used to guide the selection of study variables, data analysis, reporting, and discussion of the study findings. The Ferrans Conceptual Model [S1 Fig] was chosen because the study explored areas that are typically encompassed by the construct of HRQOL such as physical health, emotional well-being, social well-being, education/employment, and environmental factors. The Ferrans Conceptual Model highlights the influence of health-related factors, as well as characteristics of the individual and the environment, on individuals’ quality of life, making it a useful model for guiding research in YALPH since their well-being can be directly affected by both individual factors and environmental characteristics.

### Study design, setting, population and sample

This was a cross-sectional study using in-depth interviews with YALPH (aged 18–27 years) who were enrolled on ART at Botswana-Baylor Clinic in Gaborone, Botswana. The patients enrolled on ART at the Botswana-Baylor Clinic lived in Gaborone and surrounding districts; thus, they were drawn from diverse geographical and socio-economic environments. The data was collected from April to July 2019. The maximum variation sampling method was used to select information-rich cases representing variation in socio-demographic and clinical characteristics. All of the YALPH identified through the sampling approach agreed to participate in the study. The final sample size of 45 YALPH was determined by data saturation. To track and monitor saturation, the researchers combined sampling, data collection, and data analysis instead of treating them as separate stages in a linear process [36]. The researchers conducted four sequential analysis rounds following each set of ten in-depth interviews until no new information or themes emerged. Saturation was determined subjectively by the researchers after the completion of forty in-depth interviews. However, five additional interviews were conducted to substantiate the previous interviews.

### Inclusion and exclusion criteria

Participants were included if they met the following criteria: (1) age (18–30 years), (2) evidence of perinatal HIV infection in the patient’s medical records (including documented HIV-positive results, HIV-positive diagnosis in early childhood (0–8 years), having a mother with documented HIV infection, and no evidence of other modes of HIV transmission), and (3) informed consent to participate in the study. The YALPH who were acutely ill or had evident cognitive dysfunction or disabilities were deemed unable to participate and were excluded from the study.

### Data collection process

Respondents were asked to provide basic social-demographic information (e.g., age, gender, marital status, education level, employment status, place of residence, living arrangements, having any children). Clinical information was abstracted from patients’ medical records maintained at Botswana-Baylor Clinic. The researchers developed and used a semi-structured in-depth interview guide that contained open-ended questions on perceived challenges, coping strategies, and what should be done to improve the wellbeing of YALPH. The development of the in-depth interview questions was based on a detailed literature review on the challenges and coping strategies of YALPH as well as consultation with health care workers (HCWs) at the Botswana-Baylor Clinic. The interview guide was written in English and then translated into Setswana, the participants’ native language. The interview guide was pilot-tested with ten conveniently selected YALPH at the Botswana-Baylor Clinic using a cognitive interviewing approach. The in-depth interviews were conducted by two researchers (G.K. and a trained research assistant), which enabled uniformity of approach since only two interviewers were involved. The in-depth interviews lasted between 30 and 45 minutes on average.

### Study ethical considerations

Ethical approval was granted by the Institutional Review Board of Botswana-Baylor Clinic (BBCCOE/14) and the Health Research Development Committee of the Ministry of Health (PPME-13/18/1 Vol. VII (318). Written informed consent was obtained from each participant before the interview.

### Data management and analysis

Basic statistical analyses were performed to compute frequencies and percentages from socio-demographic and clinical data about the in-depth interview participants to provide context for the perspectives presented in the study. The in-depth interviews were audiotaped and then transcribed verbatim. Qualitative content analysis was used to summarize and systematize the in-depth interview data following the steps described by Hsieh HF and Shannon SE [37], which include identifying relevant themes and categorizing them into main categories and subcategories. This was done by examining, interpreting, and categorizing the participants’ narratives as challenges, coping strategies, and recommendations for improving YALPH’s wellbeing. The data generated by in-depth interviews showed very close similarities in participants’ experiences and world views, making it easy to categorize.

Next, the categories that were related to the challenges faced by YALPH were mapped to the Ferrans Conceptual Model of Health-Related Quality of Life [35]. The categories that were related to the coping strategies were mapped to the 14 scales of the Brief COPE Inventory [26,38]. The Brief COPE Inventory has 14 subscales grouped under adaptive and maladaptive coping strategies [S2 Fig]. In this study, the Brief COPE inventory was not used to collect data or directly assess the coping strategies of YALPH. The 14 scales of the Brief COPE were used to guide the categorization of the coping strategies reported by YALPH in the in-depth interviews. The coping strategies reported by the participants in response to questions 13 and 14 in the [S2 File] were linked to a particular scale and consequently classified as adaptive or maladaptive. We quantified the frequency of participants who reported coping strategies related to each of the 14 scales in order to highlight patterns in coping strategies [39]. In all cases, extensive quotations to be used in reporting the results were identified to keep the issues expressed by the participants more succinct than abstract.

We used the computer software NVivo to support data analysis. Two independent researchers (G.K. and a research assistant) categorized the same ten transcripts. Inconsistencies were resolved through discussion. G.T.T. and a social worker with extensive experience in qualitative research (who was not part of the research team) independently reviewed and validated the themes, categories, and subcategories. The remaining transcripts were categorized by two researchers (G.K. and G.T.T.). The study reporting was guided by the consolidated criteria for reporting qualitative research (COREQ) [40].

### Trustwothiness

Various strategies were used to ensure the trustworthiness of the study [41]. Credibility was enhanced by the fact that study data was collected from the same site, which is Botswana-Baylor, and primarily by the two researchers (G.K. and a research assistant), making it possible to authenticate the data. Furthermore, member checking or participant validation was used with a group of fifteen YALPH who participated in the study. The use of member checking or participant validation entails testing the data, analytic categories, interpretations, and conclusions with representatives of the group from which the data were originally obtained [41]. In this study, member checking was used to ensure that the results, interpretations, and conclusions were consistent with the participants’ personal experiences and understanding, as well as to allow them to challenge what they perceived as wrong interpretations of their perspectives regarding the challenges they faced and their coping strategies. All the YALPH who participated in the validation exercise agreed with the study’s findings, interpretations and conclusions. To ensure confirmability and dependability, the researchers took extensive field notes shortly after each in-depth interview to maintain an audit trail. The field notes allowed the researchers to reflect on the interviews and decrease preconceived perceptions and to gain a less biased interpretation of the information generated by the study. Furthermore, the Botswana-Baylor research team reviewed all aspects of the study including the study proposal, data collection tools, data storage, as well as the study findings and report.

## Results

The socio-demographic and clinical characteristics of the participants are shown in Table 1.

**Table 1:** Socio-demographic and clinical characteristics of the participants (n = 45).

Count (percentage)		
**Gender**		
Male	21(46.70)	
Female	24(53.30)	
**Highest level of education attained**		
Primary	2(4.44)	
Secondary	19(42.22)	
Tertiary/post secondary	24(53.33)	
**Marital status**		
Living as married	3(6.67)	
Single	42(93.33)	
**Living arrangements**		
Family	33(73.33)	
Rented	10(22.22)	
Boarding school or institutional care	2(4.44)	
**Occupational Status**		
In school	11(24.44)	
Employed	24(53.33)	
Unemployed	10(22.22)	
**Young parents**		
Female	6(13.33)	
Male	2(4.44)	
**Duration on ART (range in years)**	11.6–19	
**Viral load (copies /mL)**	Suppressed (<400)	Unsuppressed (>400)
	40(88.90)	5(11.10)

This study explored the YALPH’s challenges and coping strategies. The challenges are presented first, followed by coping strategies.

### Challenges faced by YALPH

The challenges reported by YALPH were linked to five domains of the Ferrans Conceptual Model: biological functions, symptoms, functional status, characteristics of the individual, and characteristics of the environment. A summary of participants’ reported challenges and their perspectives on how to address them are presented in [S1 File].

### Biological functions and symptoms

The majority of the participants in this study had suppressed VL, were clinically stable on ART, and perceived themselves to be in good health. A few participants had mental health problems (depression, stress, trauma) for which they were seeing a psychologist and or receiving treatment. Others reported chronic diseases (such as asthma, heart disease, epilepsy, and allergies) for which they were receiving treatment and were well-controlled.

#### Problems with ART

Most of the participants (35) did not have any problems with ART because they had been on it for many years. Secondly, the majority of participants reported being on once-daily ART regimens that were convenient and had few or no side effects. The participants were well aware of the therapeutic benefits of good ART adherence as well as the risks associated with poor adherence. Furthermore, participants were motivated to take their medications properly in order to stay healthy and physically fit in order to attend school, work, care for their children (for parents), and pursue their life goals. The majority of participants were able to manage their medication on their own. Participants’ main sources of treatment support included their parents, close family members, HCWs, trusted friends, and peers.

*"I have been on ART since childhood*, *and there are no difficulties that I am encountering*.*"* 21-year-old male

*"Now that I have a job and my space and my things*, *I feel like my life is okay*, *and I need to take my medications well for me to remain physically fit and to continue working*.*"* 26-year-old male, university graduate, employed

However, some participants, particularly those who had unsuppressed VL, reported occasional or longstanding problems with ART adherence. Poor ART adherence was associated mainly with the fear of stigma among YALPH living in environments with limited privacy as well as those in romantic relationships where their HIV status remained undisclosed. Other factors reported as contributing to poor ART adherence included a lack of treatment support from family members, treatment fatigue, and negative attitudes toward reliance on medications and treatments. To address treatment-related challenges, participants recommended adherence camps for YALPH and phone-based programs to remind them to take their medications and connect them to peer support networks.

*"The problem started in tertiary school*, *where we shared rooms*. *Every semester I shared a room with a different roommate*. *One semester I shared a room with a roommate and I didn’t tell him about my situation*, *so I didn’t take my medicine well*. *Sometimes I forgot to take my medicines after procrastinating*. *My parents spoke with the Dean of Students*, *who knows my situation*. *He helped me get a single room*, *making it easier for me to take my medicines*. *"* 24-year-old male

### Functional status

#### Physical functioning

**Disabilities and impairments:** The majority of participants perceived themselves to have good physical functioning. However, some participants reported physical disabilities, visual and hearing impairments, and learning difficulties that affected their physical functioning, school performance and attainment, and employability. Four participants carried exemption cards from the Office of the President (Disability Office) to ease access to public amenities such as transportation, healthcare, and other services. The participants recommended early diagnosis and intervention for disabilities, impairments, and learning difficulties.

*"I have a serious hearing problem which was not identified until I was in junior secondary school*. *In class*, *I had to sit next to the teacher or I wouldn’t hear*. *I was taken for assessment*, *but I did not get help until I had completed senior secondary school*. *"* 23-year-old female

*"I had a very serious attack of meningitis in childhood*, *which affected my nerves and vision*. *I have been told by the eye specialist that my vision problems cannot be corrected by lenses*. *Poor vision has impacted my life*, *including school and home activities*.*"* 21-year-old male

**Dissatisfaction with body image and appearance:** Some participants were dissatisfied with their body image and appearance due to short stature, low body weight, prominent skin conditions (scars, flat warts), and physical disabilities, which they associated with the effects of HIV and related medicines. Dissatisfaction with one’s physical appearance and a negative body image drove some participants to isolate themselves in order to avoid being stigmatized because of their body structures or features. Participants recommended that HCWs recognize body image issues among YALPH and counsel them to boost their self-image. In addition, the YALPH should be encouraged or supported to eat nutritious food and exercise regularly in order to build their bodies, remain physically fit, and improve their body image.

*"I have this thick scar on my face that I have had since childhood*, *and people always want to know what happened*. *It is a bit better now in senior secondary school because the students there are older and they mind their own business*.*"* 18-year-old male

*"I have lipodystrophy*. *I requested permission to wear pants in school instead of skirts in the summer because other students were making fun of my small legs*.*"* 18-year-old female

#### Psychological functioning

**Fear and uncertainty about the future**: The majority of the participants in this study had a positive outlook on the future, including being in good health due to ART, completing school successfully, finding jobs or establishing businesses, and being financially independent; and getting married and having children. However, some participants expressed worries about the future, such as unemployment due to their poor school performance and attainment; potential stigma in workplaces if people got to know about their HIV status; fears and worries about transitioning out of institutional care; and worries about financial independence after the death of their parents or caregivers. There were also worries about finding an accepting partner and telling them about their HIV status if they wanted to get married or have children.

Some participants felt that their lives were entirely dependent on medicines, and if the medicine was unavailable for any reason, they would die, as described by two respondents:

*"Our lives are very vulnerable due to this HIV*. *Some of my peers have died*. *I keep asking myself whether I will live a long life*." *20-year-old male*

*"I have a fear of the future without ART because my life is entirely dependent on medicines*. *If for some reason I don’t have the medicines or they cause side effects and don’t work anymore*, *what will I do?" 20-year-old female*

*"I am always dependent on other people*, *such as my parents*. *I face an uncertain future with HIV*, *especially when my parents are gone*. *Will I manage without them*?*"* 22-year-old male

The participants recommended that YALPH should be counseled and assessed on how satisfied they are with their lives. And if they are not satisfied, they should be supported in developing goals for the future and strategies for achieving them. Schools, HCWs, and other service providers should provide YALPH with information and support in accessing government and private sector youth empowerment programs in order to improve their well-being and give them hope for the future.

*"Engage with us young adults more*. *Inform us about the opportunities available outside of the clinic*, *both in the government and the private sector*. *Find days when we can all sit and encourage each other about the future*.*"24-year-old male*

**Grief and bereavement:** Some of the participants reported grief and bereavement over the deaths of their parents, siblings, and other close family members. The death of a parent was reported by 19 participants and was a result of HIV-related causes. Other relatives’ causes of death were not disclosed. The age at which the participants lost their parents ranged between 2 and 21 years. The impact of parental loss on the lives of YALPH was significant and long-standing. Those who had lost their mothers spoke of the loss of a strong emotional attachment typical of mother-child relationships, while those who had lost a father reported a lack of a father figure in their lives. Many participants felt that living with HIV would have been easier if their parents were still alive. Other challenges that participants faced as a result of their parents’ deaths included lack of ART adherence support, financial stressors, property-related family conflicts, and some young mothers lacking childcare support when they wanted to find work or return to school. Many YALPH had kept their grief to themselves and had not shared it with anyone.

*"My mom passed away in 2013*. *She was the only person who encouraged me to take my medication and provided everything I needed*. *During this year’s festive season*, *I was home alone and overcome by grief*. *The stress of losing my mother has affected my life*, *including my adherence to medication*.*"* 24-year-old male

*"I have a long story to tell about my life*. *I have only experienced the rough side of life*. *My mother is dead*. *My loving grandfather and grandmother are also dead*. *In my family*, *every fifth year a close family member dies*. *I am always worried that I may be the next to die*.*"* 23-year-old female

**Concerns and worries about disclosure:** The majority of participants found it difficult to disclose their HIV status to other people. Disclosure to a romantic or sexual partner was the most difficult thing for all participants, regardless of whether they had ever disclosed to a partner or not. As a result, some of the YALPH were engaged in relationships with partners to whom they had not disclosed their HIV status. The most frequently reported reasons were a lack of trust in the person with whom they were in a relationship and fear of rejection and violence. Those who had not disclosed to their partners described the stress of living in secrecy, as well as the difficulty of taking medication and attending medical appointments. Nevertheless, participants who had not disclosed to their partners reported using condoms all the time to reduce the risk of HIV transmission.

*"My biggest challenge is keeping the secret and having to hide my status from my boyfriend all the time*. *It is stressful when I have to come up with excuses for coming to the Botswana-Baylor Clinic for a check-up*, *medication refill*, *or to attend Young Adults’ Support Group meetings*. *"* 25-year-old female

Some participants (11) had been rejected after disclosing their HIV status to a sexual partner. A few participants reported staying in abusive relationships after disclosure because they were afraid the partners would disclose their HIV status to other people if they left. Participants recommended peer support and couple counseling to help YALPH deal with disclosure fears and worries.

#### Social functioning

**Family relationships**: The majority of the participants described their family relationships as healthy, caring, and very supportive. Families provided emotional support, ART adherence support, financial support, general encouragement, and a safe home environment, which helped the participants cope well with HIV and other stressors. On the other hand, some participants reported stressful family relationships, including lack of ART adherence and financial support, violence and abuse, property-related conflicts, stigma and discrimination, and family members disclosing their HIV status to other people without their consent. Some participants felt they had no one in their family to turn to when they needed help. Others felt they lived with people who did not understand their situation and that everyone was pushing them too hard. Participants recommended that HCWs should provide counseling to promote harmonious family relationships and equip YALPH with problem-solving and conflict-resolution skills.

*"I am grateful to my family for providing me with everything*. *However*, *they should try to understand me instead of blaming me for the little things that I do*. *My sister says I’m old enough to know why I should take my medicines properly*. *I live with people who have no idea what I am going through*. *It is very tough*, *but no one understands*.*"* 20-year-old female

**Friendships and peer relationships:** Some participants reported having affectionate and supportive friendships and peer relationships. In most cases, these were schoolmates, churchmates, or peers whom they met at Botswana-Baylor Teen Clubs, Young Adult Support Groups, and Young Mothers Support Groups. Peer support groups provided information, emotional support, encouragement, and forums for personal and intimate disclosure of experiences, thoughts, and ideas leading to better coping and adjustment with HIV. In contrast, many participants reported that due to the fear of stigma and discrimination, they didn’t have friends, preferred to be alone, avoided visiting or attending social events, and were only friends with close family members. The participants recommended that YALPH be linked to online and other peer support networks.

*"I still haven’t found a best friend*, *but some of my good friends I met them at the Botswana-Baylor Teen Club and the Young Adults Support Group*. *Some are older than me*. *They help me out when I am in need because they understand my situation*. *When I tell them about my problem*, *they respond in the way I want them to*, *unlike someone who tries to understand but doesn’t understand to the full extent*. *"* 25-year-old male

**Sexual and romantic relationships:** The majority of participants (40/45) had been in a relationship at some point in their lives, and more than half (28/45) were in a relationship at the time of the study. However, many participants believed that growing up with HIV had negatively affected their ability to form and maintain romantic or sexual relationships. Fear of disclosing their HIV status to sexual partners was the most frequently reported barrier to romantic relationships.

*"I am depressed all the time and on anti-depressants*. *The virus controls*. *It controls the time I have to be home; I can’t drink alcohol like other youth; and I can’t have sex when I need it*. *I feel totally out of control*.*"* 21-year-old female

The participants recommended that YALPH be given in-depth education on sexual health and relationships; that YALPH be encouraged to bring their partners to the clinic for HIV testing or counseling; access sexual health services; and that discordant couples be helped to access pre-exposure prophylaxis (PrEP).

#### Characteristics of an individual

**Poor academic performance and attainment:** Many participants reported poor academic performance and attainment, which they attributed to learning difficulties, visual and hearing impairments, and illness (particularly in childhood). Some participants did not expect to survive to adulthood, so they did not study hard in school. Poor academic performance and attainment resulted in feelings of inadequacy and worthlessness, as well as fears and uncertainty about the future. The participants with learning difficulties recommended that there should be an alternative school system that is less academic and focused on vocational or practical skills development that also assures success in life for young people who are not academically competent.

*"Since primary school*, *I have always performed poorly*, *however hard I try*. *I am always desperate and frustrated by my grades*. *Sometimes I hate myself for that*. *I am a slow learner*, *but in school we are not given the chance to learn slowly*. *I prefer to do some training in a technical school and start working*." 18-year-old male

**Unemployment:** Only nine participants were employed in full-time professional jobs, which gave them financial independence and a sense of self-worth. However, fifteen participants worked in low-paying jobs with long working hours, heavy workloads, or poor working environments. Those participants wished to be helped in accessing vocational or tertiary-level training to gain technical skills and find beneficial employment. Ten participants were neither in school nor working due to a lack of post-secondary education qualifications or job scarcity. The unemployed participants had financial stressors and could not meet their basic needs. Participants advocated for increased access to vocational training, job skill development, employment, and entrepreneurship opportunities.

*"Because of my poor performance during my school years*, *I did not make it to tertiary, and I do not have the qualifications needed to find a job*. *I am doing nothing, which is why I am always bored at home*. *I need access to job opportunities where I can use my own hands, such as selling in shops or working on construction sites*. *I am physically fit and can work with my hands*. *I need to make some money to support myself while also sponsoring myself for a course*.*" 24-year-old male*

**Parenting challenges:** Eight of the participants were parents (6 females and 2 males). The narratives show that parenting was gratifying, and it motivated young parents to take their medicine well in order to stay healthy and provide for their children. Some of the young parents were satisfied with the child care support provided by their family members and partners. The Botswana-Baylor Young Mothers’ Support Group was cited as a beneficial forum for knowledge sharing and emotional support from peers. Some of the mothers were supported through government-provided services, such as public works employment (Ipelegeng) and nutritional support for their children through Child Welfare Clinics in their communities.

However, some mothers were unemployed, and they reported challenges with single parenting, unsupportive fathers of their children, and in some cases, a lack of support with child care when they wanted to return to school or find employment. Two young mothers with HIV-positive children felt guilty and blamed themselves for transmitting HIV to their children. The young mothers faced challenges managing their own and their children’s health. Those mothers said they received emotional and other support from family members and partners, as well as peer support at the Botswana-Baylor Young Mothers’ Support Group. However, according to the two young mothers’ narratives, the guilt of passing the virus on to the child persisted.

*“I was pregnant and was shy about being seen by people at the Botswana-Baylor Clinic*. *As a result, I stayed away*. *My seven-month-old son is also HIV positive*. *I do feel a little guilty." 27-year-old female*

*"I feel guilty and blame myself for not taking my medications properly and infecting my son*. *But I try to ignore those feelings*. *I’m afraid my son will blame me for infecting him, and living through that situation worries me*.*" 18-year-old female*

The young parents advocated for increased access to vocational and tertiary education, as well as employment opportunities, in order to improve their financial capacity to provide for their children. The young mothers proposed the expansion of peer support groups for the exchange of knowledge and experience in parenting. The two young fathers called for male-focused sessions to teach them about fatherhood and parenting. The young fathers perceived themselves as good parents in general, but they recommended that all young fathers be recruited into fathering programs to develop the social skills needed to effectively fulfill their roles as parents, such as improving their ability to manage relational stress and conflict with their partners and how to nurture and provide for their children.

**Transitioning out of institutional care**: Three participants who had aged out of institutional care had received exit preparation, which included counseling on reintegration into their families of origin or establishing independence in the community. They did, however, face many challenges such as family conflicts, processing national identity cards, unemployment, financial stressors, and other things for which they had not been prepared.

*"Things such as a national identity card are things you don’t pay a lot of attention to until you need them*. *The other thing is the lack of preparation for dealing with family issues*. *My sister and I struggled and still struggle to claim back our parents’ house and property from our uncle and his family*. *Nobody helped me prepare for it*. *"* 27-year-old female

The participants recommended that after exiting institutional care, youth should be assigned adult mentors or someone they are generally able to engage with who will assist them in navigating various systems in order to access needed support services.

#### Characteristics of the environment

**Fear of stigma and discrimination:** The narratives show that the majority of participants had constant fear and worry about how others would react upon learning of their HIV status. As a result, the participants felt the need to protect themselves by not disclosing their HIV status and/or isolating themselves from social gatherings, friendships, and intimate relationships. The majority of the participants had the fear of being stigmatized despite never experiencing an act of stigma because they were physically fit and only close family members and a few trusted people knew their HIV status. Some participants, however, had experienced acts of stigma, heard negative comments, or witnessed discrimination against other PLWH. Participants recommended a range of stigma reduction strategies, including integrated service delivery at health facilities, policies to ensure YALPH privacy in residential facilities and boarding schools, and increased stigma-reduction education targeting the youth.

*"When they see me going to the clinic regularly*, *I begin to imagine that they know*, *or they may guess right*. *At one point*, *I stopped coming for treatment for five months because there were too many people who knew me at the Botswana-Baylor Clinic*. *"* 21-year-old female

**Financial stressors:** The majority of participants identified the lack of money to meet their basic needs as a major stressor. Financial problems were attributed mainly to limited family resources, unemployment, low or unreliable wages, lack of capital and ideas to start businesses, as well as disabilities and impairments. The participants also reported that their families considered them to be old enough to work and be financially independent. Financial insecurity affected some participants’ ability to meet transportation costs to the clinic for medical appointments, resulting in occasional gaps in medication adherence. Financial constraints also made it difficult for some participants to go back to school to retake exams or enroll in vocational or tertiary level courses if they did not qualify for government sponsorship. Others reported food insecurity, family conflicts, limited access to the internet, and other sources of essential information and social networking. The participants advocated for initiatives to empower YALPH to access employment opportunities and skills for generating and saving income.

*"There are too many conflicts in my family*, *and they are all about money*, *spending*, *and eating*. *My mother says that I am now old enough to care for myself and that I should stop acting like a child*. *She wants me to go out and be like other young people*, *taking care of myself with everything I need*. *I need a job*, *but I cannot find one*.*"* 23-year-old male

### Coping stategies

The participants used a range of coping strategies, both adaptive and maladaptive, to live with HIV infection and related stressors. The coping strategies reported by the participants in the indepth interviews were mapped to the 14 scales of the Brief COPE Inventory as shown in (Table 2).

**Table 2:** Summary of participants’ coping strategies.

Adaptive Coping Strategy		*Frequency
**1.**	Active coping ***(acting in response to a situation)***	(41)
**2.**	Use of emotional support ***(getting emotional support from others)***	(40)
**3.**	Acceptance***(accepting the reality of HIV infection and learning to live with it)***	(40)
**4.**	Use of instrumental support**(*seeking advice*, *help*, *or information and learning from others)***	(39)
**5.**	Planning***(coming up with a strategy about what to do)***	(37)
**6.**	Religion ***(praying and spiritual belief)s*. *Getting the strength through belief in God***	(14)
**7.**	Positive reframing (***making the situation seem more positive)***	(11)
**8.**	Humor*(****making fun of the situation*, *irony****)*	(6)
Maladaptive Coping Strategy		
**9.**	Self-distraction**(*doing something to think about it less)***	(25)
**10.**	Venting **(*expressing negative feelings****)*	(17)
**11.**	Self-blame **(*self-criticism)***	(7)
**12.**	Substance use **(*use of alcohol and other drugs to feel better)***	(5)
**13.**	Denial **(*refusing to believe that it is real or true)***	(3)
**14.**	Behavioral disengagement **(*giving up the attempt to cope)***	(0)

*The frequencies show the number of participants who reported coping strategies aligned with each of the 14 scales of the Brief COPE inventory.

#### Adaptive coping strategies

*Active-coping (acting in response to a situation)*. Almost all participants (41) were taking action and had plans to remain in control and in good health, primarily by adhering to ART and keeping their HIV status confidential. All participants had positive perceptions of the benefits of ART on their health and well-being. Choosing to keep their HIV status confidential and disclose it only to close family members and a few trusted people to avoid possible stigma and rejection from others was a major protective and active coping strategy that nearly all of the participants adopted. The quotes below demonstrate this coping strategy:

*"I started coming to Botswana-Baylor Clinic in 2004*. *Before that*, *I was in and out of the hospital and very weak*. *I never went to school because I couldn’t sit without being supported*. *After starting the treatment*, *my life changed*, *and right now when I walk down the street*, *no one can tell whether I am HIV positive or not*. *So I think ART covers the difference between someone who is positive and someone who is not positive*. *It has helped me to live a normal life*, *so ART is very important*, *and I will continue to take the medicines as prescribed*.*"* 22-years-old male

*"These medications are good because even when I am with someone who is not infected*, *there is no difference*. *There will be a difference only if I stop taking the medicines*. *This means that if I take the medicines correctly*, *I can do a lot of things and live longer than someone who doesn’t know their status*.*"* 26-year-old female

*"No one knows my HIV status*. *I don’t want them to know because they’ll start stigmatizing me*. *I have a feeling that they will look at me differently*, *which I do not want*. *There’s also the possibility that they’ll feel pity for me*, *and now I’ve told myself that I will not tell them*.*"* 21-year-old male

*Use of emotional support (getting emotional support from others)*. The majority of participants (40) said they were able to overcome their struggles, be hopeful, develop self-confidence, and adhere to ART as a result of the love, care, reassurance, encouragement, and emotional support from their family members, HCWs, romantic partners, peers, and trusted friends. The Botswana-Baylor Teen Club, Young Adults’ Support Group, and Young Mothers’ Support Group were frequently identified as group-level sources of emotional support, allowing them to freely express their feelings and share experiences with peers, all of which resulted in improved coping and well-being.

*"I disclosed my status to my two childhood friends*. *I sat them down and I told them that I am HIV positive*, *and according to the doctor*, *I contracted it from my mother*. *They told me right away that I was not alone*. *They gave me support*, *and they have been there for me ever since*. *Even if I text them or message them on Facebook or WhatsApp that I have a headache*, *they will tell me to consider my health first*. *A minor problem should be taken to the doctor*." 21-year-old female

*Acceptance (accepting the reality of HIV infection and learning to live with it)*. The majority of participants in this study (40) had accepted their HIV status. What helped them accept their HIV status included the strong support from parents and close family members and the education and counseling provided by HCWs. Some participants said they didn’t believe they were HIV-positive, so they re-tested, confirmed their HIV status, and then started taking their medicines as prescribed. Some participants accepted after falling very sick, their VL having been very high, and their immune system being extremely weak as a result of poor ART adherence or defaulting from treatment.

"I did my own testing because I didn’t believe I was HIV-positive. I was told by healthcare workers, but I never saw the results. I re-tested at a group called BORNUS, and my HIV status was confirmed. After that, I started to take the medicine properly. " 26-year-old female

*Instrumental support (seeking advice*, *help*, *or information and learning from others)*. The narratives showed that the majority of participants (38) also coped by seeking and receiving advice, help, or information from family members, HCWs, friends, peers, and church leaders. The participants had a very strong relationship with the HCWs at the Botswana-Baylor Clinic, who were always available to provide ongoing advice and information on how to deal with both clinical and psychosocial challenges. The Teen Club, Young Adult’s Support Group, and Young Mothers’ Support Group were cited as highly effective sources of instrumental support, particularly the bonding and learning from other youths who had successfully navigated similar challenges with them.

*"I sometimes need advice from someone who has the same problem or status as me*, *on things like medication adherence*, *because they understand what I am going through*.*"* 18-year-old male

*Planning (coming up with a strategy about what to do)*. The majority of participants (37) coped with HIV infection through self-determination and planning. Essentially, participants had adjusted to being HIV positive and had taken a stand in life to take care of themselves and live a good and healthy life. Participants spoke of persevering, considering available opportunities, being willing to act, strategizing, and taking steps to deal with their problems to improve their well-being and achieve future goals. For example, those who were out of school and unemployed planned to pursue vocational training in order to acquire relevant job skills and increase their employability. Others intended to use government-funded youth empowerment schemes to start businesses and achieve financial independence.

"I have big dreams that I want to accomplish in my life. Right now, all I can think about is me and school. In the past, I didn’t have time to focus, so I failed my exams, but now I have a second chance. School is the only way for me to get somewhere. I am also taking driving lessons because I want to get my driver’s license by the end of this year." 21-year-old female

*Religion (prayer and spiritual beliefs or obtaining strength through faith in God)*. Spirituality, religion, and faith in God were mentioned by some participants (14) as sources of hope, strength, and encouragement in coping with HIV-related challenges. Those participants attributed their good health, longevity, school attainment, and all other accomplishments to God. The narratives showed that those participants put their trust in God to help them manage the challenges in their lives, while others attended church on a regular basis and received spiritual and emotional support from their pastors, churchmates, and youth groups. Others found religion to be a source of healing from the anger and blame they had towards their biological parents for infecting them with HIV and helping them have a positive outlook on life. When others had illness-related fears and worries, they turned to prayer or listened to gospel music.

*"I am a child of God because I am a Christian*. *If you are a child of God*, *you should always walk with power and hope*, *and nothing should steal your peace*. *My God has given me peace*. *It is within me*, *and it is my responsibility to put that peace to use*. *I do that all the time*. *That is why I can now say that my life is settled*. *I can live with the problems in my family*, *and I can take my medicine well*.*"* 22-year-old female

*Positive reframing (making the situation seem more positive)*. Some participants (11) said they coped by replacing negative, self-defeating thoughts with positive, affirming feelings. For example, some participants felt advantaged because they were connected to the Botswana-Baylor Clinic, were in contact with HCWs, and their health was continuously monitored as compared to individuals who only used health care services once in a while. Some participants even perceived long-term beneficial consequences of their condition. Being HIV positive motivated some of the YALPH to work harder and finish school successfully, avoid negative peer influences such as drug and alcohol abuse, eat nutritious meals in order to stay healthy and strong, be successful in life, and prove those who doubted their abilities wrong.

*"I learned a lot of things that most people my age have not experienced*. *Some of them get to experience them later in life*, *but I got to experience a whole lot of things when I was young*. *I realized very early that this was a condition I couldn’t change*. *I told myself I wanted to live and change my living situation*. *So I set my life goals very early and very high*. *I decided to take my medicine as prescribed and come to the clinic for regular check-ups*.*"* 19-year-old female

*Humour (making fun of the situation*, *irony)*. A few participants (6) used humor as a coping strategy. They reported that they were stressed when they first learned of their HIV status. However, over time, they accepted it, and they were able to cope by making fun of and joking about their situation.

*I learned about my HIV status 12 years ago*. *My entire world came crashing down that day*. *Later*, *I came to grips with who I was and what was going on inside of me*. *Now it is okay; I have learned to live with it*. *I view it in a childish way*, *as if I’m like a robot that always has to recharge its batteries*. *Every time I take my pills*, *I view it that way*, *and it doesn’t seem so sad anymore*.*"* 23-year-old male

#### Maladaptive coping strategies

*Self-distraction (doing something to think about it less)*. Many participants (25) engaged in avoidance coping or self-distraction. Participants either become engrossed in school, work, church, social media, sports, reading, or watching television and listening to music to think less about their condition. Others simply avoided talking about HIV as much as possible. Participants reported that apart from reminders to take medication and go for check-ups, discussions about HIV were avoided, even with close family members.

*"My school is very interesting*. *I like to sit around and chat with my friends*. *I enjoy going to the fellowship and the library*, *as well as playing chess and attending sporting events*. *It keeps me busy and prevents me from dwelling on my situation*.*"* 21-year-old male

*Venting (expressing negative feelings)*. Some participants (17) were resentful and angry at their biological parents for HIV infection. Others expressed negative feelings towards family members who stigmatized, discriminated against, or were violent towards them. According to the participants, venting was not only a way to release unpleasant feelings but was also a means to get an effective response from others (e.g., biological parents, family members, and unsupportive fathers of their children). Some participants were angry because they believed HIV infection limited, restricted, and controlled everything important to them, preventing them from enjoying life as other HIV-negative youth did. Some participants used alcohol to calm their anger, while others turned to religion.

"*For the past ten years*, *since I was informed about my HIV status*, *I have been angry*. *I have always wanted to know why*, *of all my six siblings*, *I’m the only one who is HIV-infected*. *I sometimes spend nights out and drink alcohol*, *which helps cool my anger*. *I do this primarily to anger my mother for infecting me with HIV*. *I don’t want to see*, *hear*, *or deal with her anymore*. *"* 22-year-old male

*"Having this virus has imposed significant restrictions on me*. *It dictates what I drink*, *when I eat*, *when I go home*, *and the kind of woman I will marry*. *I had a dream of being a soldier*, *but that will never come true because there are restrictions on HIV-infected people joining the military*. *HIV limits choices in life*.*"* 22-year-old male

*Self-blame (self-criticism)*. Some participants (7) blamed themselves for their difficult situation. For being perinatally HIV-infected while their siblings were not; failing to adhere to ART and having unsuppressed VL; not studying hard and failing school exams; failing to take their medication well and giving birth to an HIV-infected child; or being generally unlucky.

*"I am the unlucky child when compared to my siblings who are not on ART*. *I have a strong belief that something is wrong with me*. *I don’t understand it*. *Everything I try fails*. *I am constantly worried about what will happen to me in the future*. 19-year-old female

When asked how they could be helped to deal with the anger and negative feelings towards themselves and others, participants proposed that HCWs should provide more counseling and facilitate open communication between parents/caregivers and the YALPH in order to address any unanswered questions that they (YALPH) may have.

*Substance use (use of alcohol and other drugs to feel better)*. To cope with HIV infection, a few participants (5) drank alcohol to think less about their condition. Alcohol abuse was also associated with unemployment and idleness.

*"I am unemployed*, *and I am bored all the time*. *So some evenings and on weekends*, *I go out partying with my cousins and friends*. *Taking alcohol helps me forget about my problems*. *But I do not drink too much because alcohol interferes with medication*." 24-year-old male

*Denial (refusing to believe that it is real or true)*. A few participants (3) were still in denial or had doubts about their HIV status. For example, one 18-year-old male who had been informed about his HIV status five years before, was still processing the results and had not yet accepted them. In addition, two participants were tested for HIV when they were very young. They had always been healthy, and they had never seen their HIV test results or documentation. To them, there was no evidence of being HIV positive. However, all three participants followed the doctors’ instructions, took their medicines as prescribed, and their VL was suppressed:

*"I can’t describe how I feel about being HIV positive*. *It’s not the kind of thing you want to think about all the time*. *You try to ignore it and don’t think about it*.*"* 20-year-old male

When asked what support they needed to deal with denial, participants recommended that HCWs should provide detailed information about HIV and AIDS, answer any questions that YALPH may have about their condition, and refer them for a re-test if necessary.

## Discussion

The majority of YALPH in this study perceived themselves to be in good health and physical functioning. The findings extend the literature demonstrating good physical functioning in adequately treated young people living with HIV [34,42–44]. These findings are encouraging because good health underlies the ability to be successful in education, employment, social relationships, and other young adult roles. This finding emphasizes the importance of expanding optimal HIV/AIDS treatment and care programs to optimize the health and wellbeing of YALPH.

However, despite the good health outcomes, some YALPH had mental health problems or chronic illnesses for which they were receiving treatment and were well managed. These results are consistent with results of various studies conducted in other settings globally which suggest that YALPH are at high risk of mental health disorders including depressive disorders, anxiety disorders, substance use issues, post-traumatic stress disorders, and psychiatric symptoms compared to their HIV-negative peers [7]. Mental health disorders and psychiatric problems among YALPH have been associated with poor ART adherence, viremia, and poor health outcomes [7,9]. Although mental health problems were reported by a few participants, the results imply that screening, assessing, and treating specific categories of mental health and psychiatric problems may enhance efforts to improve ART adherence, prevent poor health and improve well-being in YALPH. This will require the development of guidelines and training for service providers to timely identify, refer, or manage mental health problems in YALPH. In addition, developing strategies to effectively prevent, monitor and manage chronic illnesses in YALPH will be important [45,46].

Furthermore, some YALPH particularly those with unsuppressed VL reported challenges with ART adherence arising mainly in response to the fear of stigma. The findings are consistent with previous research which found challenges with ART adherence in YALPH leading to poor health outcomes [5–11]. Perfect adherence to ART is needed to achieve VL suppression and prevent the onward transmission of HIV to sexual partners and offspring. Therefore, youth-friendly intervention models to promote ART adherence and long-term VL suppression which were proposed by YALPH in this study (peer support groups, adherence camps, and phone-based adherence support), need to be considered by policymakers and programmers [11]. Studies in various settings globally have shown the potential benefits of phone-based interventions for ART adherence in young people living with HIV [47,48]. Therefore, adequate resources should be allocated to using mobile digital media and social networking to influence long-term ART adherence in YALPH.

Perceived stigma as an environmental factor was found to be a major challenge negatively affecting ART adherence and limiting the formation of social and romantic relationships. These findings are consistent with other studies that showed that stigma affected YALPH well-being by undermining ART adherence [49,50], reducing social support resources [8–10], and resulting in decreased general psychological health [31,51]. Therefore, diverse interventions should be adopted to target the root causes of stigma as well as specific environments where YALPH are confronted with stigma on a daily basis. The stigma reduction interventions proposed by YALPH in this study, such as integrated service delivery at health facilities, policies to ensure YALPH privacy in residential facilities and boarding schools, and increased stigma-reduction education targeting the youth, should be considered. The results highlight the need for the Ministry of Health (in collaboration with community-based partners) to increase community sensitization and education to reduce stigma and foster a supportive environment for YALPH. Research is needed to identify factors that buffer, protect against, or reduce the impact of stigma and discrimination on YALPH in order to inform interventions for this population. For example, there is a need to investigate how information technology and social media can be used to educate the youth in communities and reduce stigma against YALPH.

Another significant environmental challenge identified by most participants was limited financial resources, resulting mainly from unemployment, low-paying jobs, and limited family financial resources. Physical health was not a barrier to employment among the YALPH in this study, but rather a lack of post-secondary education qualifications and job scarcity. These findings are not surprising because young adults are traditionally expected to find employment and achieve financial independence from their parents or families of origin as a marker of becoming an adult. In YALPH, educational attainment may be impacted by non-cognitive factors such as illness, visual and hearing impairments, as well as HIV-related cognitive deficits [11,12,16,42]. These findings point to the need for targeted interventions to support YALPH in obtaining higher-level education qualifications and access to employment and business opportunities in order to generate income and become financially independent.

The Government of Botswana has established a wide array of policies and programs to increase youth employment and economic self-sufficiency. These programs include expanded access to tertiary and vocational education for vulnerable youth; entrepreneurship funding schemes; work skills development initiatives like the National Service Program and National Internship Program; and employment opportunities through Public Works Programs [17]. All those programs have a special dispensation for unemployed youth and those with special needs. As recommended by the participants, adult mentors are needed to help YALPH navigate the systems to access and utilize the educational, employment, and livelihood support schemes that are available to them.

Regarding social relationships, most of the participants had strong supportive relationships with their parents, family members, HCWs, and peers which helped them cope well with HIV. The findings are encouraging because the formation and maintenance of a wide range of social relationships and networks are central to the life course of young adults and have both direct and indirect effects on their physical and mental health [49,52,53]. These findings imply that interventions designed to improve the well-being of YALPH should use those social relationships and networks as intervention points. Future research should examine the qualities or attributes within these social relationships and networks in order to inform interventions to promote positive coping for YALPH.

On the other hand, some YALPH felt socially isolated and lacked the social support needed to cope with the challenges they faced. These findings call for interventions to routinely screen YALPH for social support needs, promote open family communication, and connect YALPH to various social support networks. As recommended by the participants, social media and other online programs should be used to identify potential vulnerabilities of YALPH and link them to supportive social networks. Special attention should be paid to the reports that many YALPH were in romantic relationships, however, their HIV status posed challenges due to the fear of disclosure, rejection, and violence. Previous research on HIV-infected youth found that the fear of disclosing their HIV status to sexual partners was a significant barrier to establishing and maintaining healthy romantic relationships and negatively affected sexual quality of life [49,54,55]. Disclosure of HIV status is critical to preventing HIV transmission to sexual partners. These findings call for interventions to encourage disclosure and the formation and maintenance of healthy romantic relationships.

This study identified a range of psychological stressors among YALPH, including long-standing grief and bereavement, dissatisfaction with body image and appearance, disclosure fears and worries, future-related worries and concerns, and stressful romantic and family relationships. The findings are significant because psychological stress can contribute to the development of mental health disorders, putting the YALPH at high risk for poor medication adherence, viremia, poor health, and low quality of life [6,7,9,12,56]. Research in other settings has shown that life stress is one of the strongest known predictors of youths’ overall satisfaction with life, both directly and indirectly, by influencing youths’ likelihood to use maladaptive coping strategies such as self-distraction or using anger, alcohol, or drugs to cope with the stress [18,47,48]. Therefore, interventions to prevent, screen for, assess, and treat the psychological stressors identified by this study are critical to improving the health and well-being of YALPH.

Transitioning out of institutional care and back into their families of origin or independent living in the community was a major challenge for some YALPH. In Botswana, the standard requirement is that youth exit institutional care between the ages of 18 and 24 years when they reach the age of majority or when they finish school [3]. There is limited information on the transitioning of young people who are aging out of institutional care in Botswana. However, studies conducted in various settings globally showed evidence of difficulties in transitioning to adulthood for former institutionalized young adults, such as unemployment, difficulties achieving financial independence, and poorer physical and mental health than other young adults or the general population [57–59]. The United Nations Guidelines for the Alternative Care of Children provide countries with guidance on preparing young people for the transition from the alternative care system to independent living [60]. The organizations releasing YALPH from long-term institutional care need to adhere to those guidelines.

Regarding coping strategies, the study found that YALPH used both adaptive and maladaptive coping strategies. However, adaptive coping strategies were predominant. The most commonly used adaptive coping strategies were active coping, emotional support, acceptance, use of instrumental support, and planning. This finding is encouraging, given the challenges associated with HIV as a chronic illness [5–11]. Strong emotional and instrumental support from HCWs, family members and peers was found to have a positive impact on other adaptive coping strategies such as acceptance, religion, and active coping. The finding about the positive impact of peer support groups on adaptive coping in YALPH is not surprising given the vital role of peer influence in the lives of young adults. Previous research has demonstrated the effectiveness of peer-led interventions in promoting ART adherence, VL suppression, and retention in care among HIV-positive youth [61–63]. The chronic disease self-management model for adults is another evidence-based healthcare approach that has demonstrated the effectiveness of peer support [64]. The findings highlight the need to expand peer-led interventions for YALPH, whether through individual or group support, community or health facility-based, in-person or virtual support models.

Conversely, self-distraction and venting were the most commonly used maladaptive coping strategies. Self-distraction is an emotion-focused coping strategy used to divert attention away from a stressor and toward other thoughts or behaviors unrelated to the stressor, with the goal of minimizing the emotional distress associated with the stressor [24]. It has been noted that short-term self-distraction may help take an individual’s mind off an intense emotion, help prevent unhealthy behaviors (such as alcohol or drug use or deliberate self-harm), and provide enough time for transition to adaptive coping strategies [65,66]. However, if self-distraction is used as a maladaptive strategy for an extended period of time, it may result in no reaction when a reaction is required to address the stressor [67]. The good thing is that all of the participants who used self-distraction as a coping strategy combined it with acceptance and used safe behaviors to distract themselves and think less about their situation (e.g., social media, reading, sports, and watching television). Therefore, it is critical that HCWs and other service providers understand how YALPH distract themselves from HIV-related distress and help them develop skills to transition to adaptive coping.

Regarding venting, the participants commonly expressed negative feelings (anger and resentment) toward their parents, family members, and the fact that they were HIV-positive. Negative feelings about HIV infection were linked to the perception that HIV restricted and controlled important aspects of one’s life, such as social interactions and career prospects. Religion and spiritual beliefs helped some YALPH to deal with their anger. This calls for religious organizations to be included in community-based support programs by providing religious counseling services to YALPH. Furthermore, because some of the participants used alcohol to cope with their anger, substance abuse prevention, screening, intervention, and treatment are critical for promoting adaptive coping in YALPH [31,68]. To deal with the negative feelings towards others, participants proposed that HCWs facilitate open communication between parents/caregivers and the YALPH in order to address any unanswered questions that they (YALPH) may have. This finding suggests that HCWs, parents, and other family members should patiently listen to YALPH and provide opportunities for the expression of negative feelings and concerns.

## Strengths and limitations of the study

This was the first study in Botswana to explore the challenges and coping strategies of YALPH. The researchers are confident that the findings will contribute to the body of knowledge on YALPH and inform policies and programs aimed at improving their health and well-being. However, the study had some limitations. First, the study’s sample of YALPH was restricted to the Botswana-Baylor Clinic, a clinical centre of excellence where the majority of patients were clinically stable on ART and had long-term access to diverse psychosocial support services. It should also be noted that the study excluded young adults who were not on ART or were very sick. The Botswana-Baylor Clinic is a large facility that serves youth from a wide geographic area, representing both urban and rural living environments. However, despite this apparent representativeness, ART sites in Botswana vary greatly in the clinical and psychosocial composition of their patients. Therefore, additional qualitative studies are required to investigate the challenges and coping strategies of unrepresented subpopulations of YALPH in diverse geographical locations. These should include young adults who acquired HIV through sexual contact or other means, those not on ART or sick or hospitalized, members of vulnerable and key populations such as youth living in rural and hard-to-reach areas, and the LGBTIQA+ community. Second, the Brief COPE inventory was used to guide the content analysis of information on the coping strategies of YALPH. To further validate the results, future research should use the Brief COPE inventory to assess coping in a representative sample of YALPH using a quantitative approach.

## Conclusion

Although the majority of YALPH in this study were in good health, they faced a number of challenges, including fear of stigma, low educational attainment, unemployment, lack of financial independence, as well as social isolation and psychological stressors. These findings call for diverse interventions by the government and other partners to prevent, screen for, assess, and manage all the challenges reported by YALPH. The results also showed that the YALPH used a combination of adaptive and maladaptive coping strategies to live with HIV and related stressors; however, adaptive coping strategies were more prevalent. Individual resilience, family, peers, and HCW support played important roles in supporting the healthy coping of YALPH. This calls for innovative interventions that educate YALPH about the benefits of adaptive coping and the disadvantages of maladaptive coping, as well as those that empower them to be resilient and seek support for instrumental or emotional reasons.

### Recommendations for practice

Results from this study have implications for the development of interventions that address the challenges faced by YALPH and empowering them to cope better with HIV:

#### Intensify national-level strategies to promote the health and wellbeing of YALPH

The MOH should intensify mobilization, advocacy, and action to promote the health and wellbeing of YALPH in Botswana. This can be done by raising awareness among policymakers, programmers, health managers, clinicians, and other service providers at institutional and community levels. National-level surveillance data on YALPH should be published periodically to inform policy and programming. The MOH, funding agencies, and all government and non-government organizations that fund programs or provide services to PLWH should, whenever possible, target YALPH.

#### Invest in the most disadvantaged YALPH

The results showed that the most challenged subgroups of YALPH included those with poor ART adherence, disabilities and impairments, those who are aging out of institutional care, those who are out of school and not working, young parents, and those with maladaptive coping strategies. Those YALPH shared several characteristics and experiences, including poor educational attainment, financial stressors, psychological stressors and lack of social support, which put them at a high risk of poor wellbeing. We advocate for deliberate action to identify and assist the most vulnerable subgroups of YALPH in gaining access to education, employment, livelihood, psychosocial and other support they need.

#### Engage YALPH in the development of policies and programs that affect them

The results of this study show that YALPH have an understanding of the challenges that they face, and they need to be consulted when decisions that affect them are being made because their input will contribute to better decision-making. For example, young mothers should participate in the development of mother-child care programs, those with disabilities and impairments should be involved in disability programming, and those who are neither in school nor working should be involved in livelihood programming. Meaningful participation will entail further developing YALPH’s skills and competencies to participate in health and wellbeing decision-making processes tailored to their changing needs.

#### Expand interventions to increase the potential for educational success among YALPH

The stressors experienced by YALH as a consequence of poor educational attainment and having limited skills with which to obtain gainful employment were profound. Poor educational performance and attainment were mainly attributed to learning problems and visual and hearing impairments. Therefore, the education sector should identify, test, and validate simple in-school neuro-cognitive screening tools to identify learners with neuro-cognitive deficits and refer them for early remediation and/or other interventions. Schools should also expand their efforts to identify and manage visual and hearing impairments and other disabilities as early as possible.

#### Focus on preventive approaches to improve the health and wellbeing of YALPH

The majority of the challenges (individual, environmental, and functional) reported by the YALPH in this study are preventable. Therefore, diverse preventive screening, education, counseling, and remediation services should be offered simultaneously, targeting the risk factors for the various challenges before they begin. Prevention services should be started in childhood and the pre-adolescent years and continue through young adulthood. As a starting point, evidence-based preventive care guidelines for YALPH should be developed. Multiple approaches should be used to implement the preventive guidelines, including educational, clinical, psychosocial, and other support services that are offered to YALPH.

#### Identify, implement, and evaluate interventions for the development of adaptive coping strategies among YALPH

Special attention should be paid to the identification, development, implementation, and evaluation of interventions that can support the development of adaptive coping mechanisms throughout childhood and adolescence and reduce the likelihood of maladaptive coping in adolescence and young adulthood. Interventions for the development of adaptive coping skills should begin early in childhood, preferably at the concrete operational stage (7–11 years), when children develop logical reasoning, including inference of cause-effect relationships and abstract reasoning [69]. There should also be training programs for HCWs, other service providers, parents and caregivers to enhance their understanding of how to promote the early development of adaptive coping styles in this population.

## Supporting information

S1 Fig Ferrans Conceptual Model of HRQOL. (TIF)

S2 Fig Brief COPE Inventory. (TIF)

S1 File Summary of challenges and proposed solutions: Perspectives of YALPH. (DOCX)

S2 File Indepth-interview guide. (DOCX)
