# Supplementary figures and images for: Challenges and coping strategies among young adults living with perinatally acquired HIV infection in Botswana. A qualitative study
Source: PLoS One. 2023 Apr 26;18(4):e0284467. doi: 10.1371/journal.pone.0284467 (PMC10132588; doi:10.1371/journal.pone.0284467)

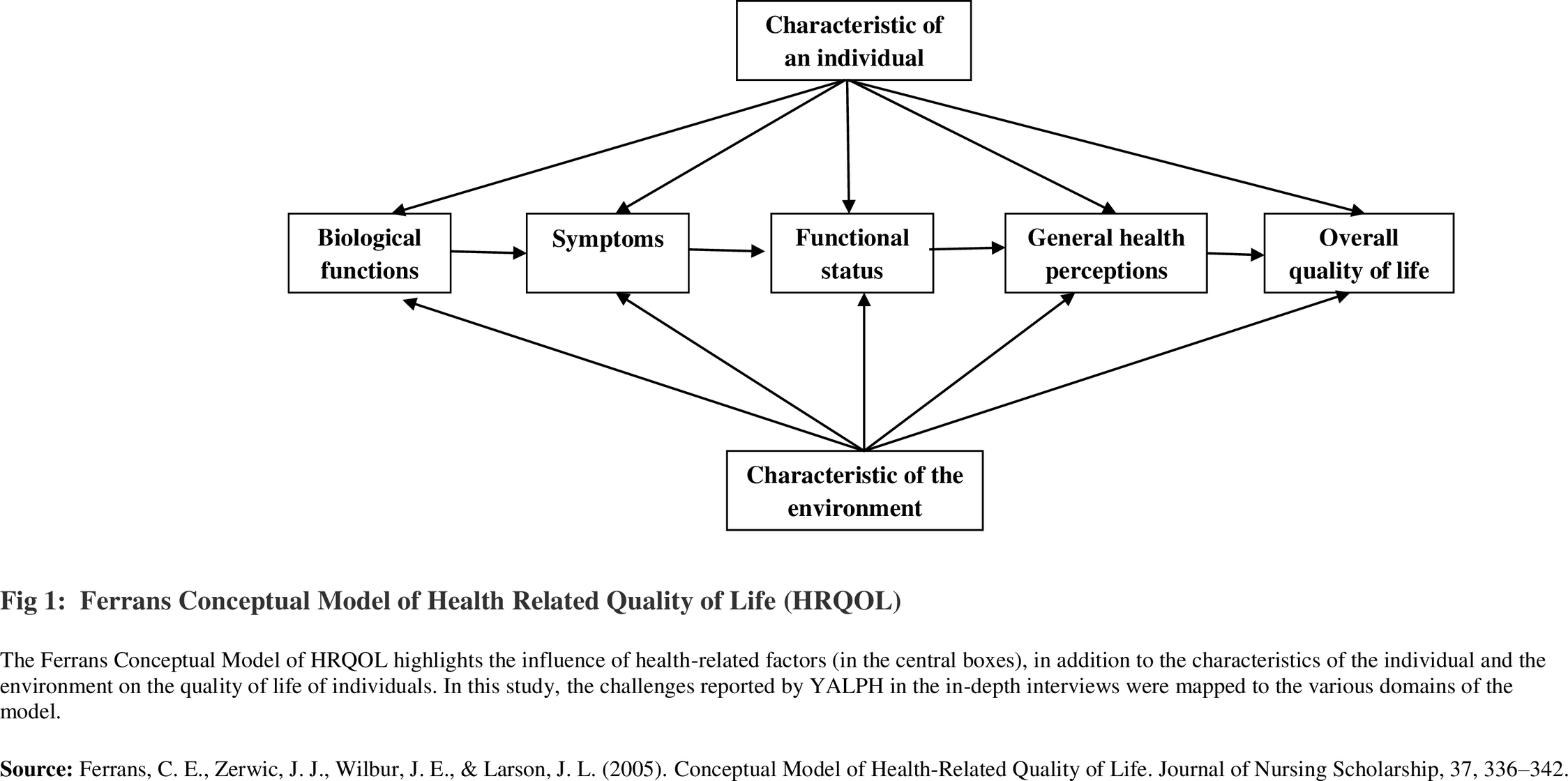

Supplement: S1 Fig — (TIF) [file pone.0284467.s001.tif]

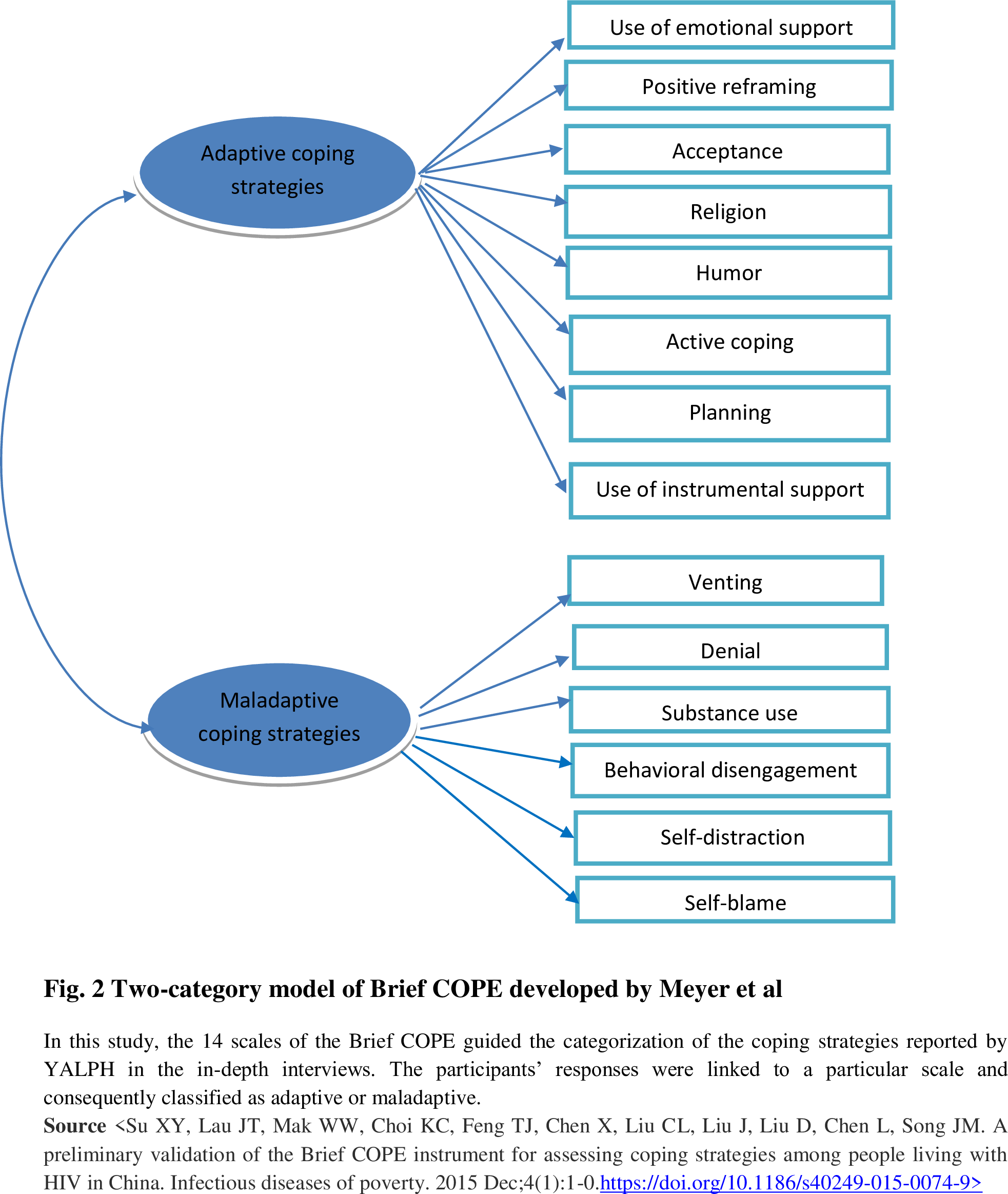

Supplement: S2 Fig — (TIF) [file pone.0284467.s002.tif]
