# Supplementary material for: Challenges and coping strategies among young adults living with perinatally acquired HIV infection in Botswana. A qualitative study
Source: PLoS One. 2023 Apr 26;18(4):e0284467. doi: 10.1371/journal.pone.0284467 (PMC10132588; doi:10.1371/journal.pone.0284467)
Supplement: S1 File — (DOCX) [file pone.0284467.s003.docx]

| **S1 FILE 1 SUMMARY OF CHALLENGES AND PROPOSED SOLUTIONS: PERSPECTIVES OF YALPH** | | |
| --- | --- | --- |
| ***DOMAINS** | ****CHALLENGES** | *****PROPOSED SOLUTIONS** |
| **BIOLOGICAL FUNCTIONS AND SYMPTOMS** | **Poor ART adherence due to fear of stigma, lack of treatment support at home, treatment fatigue, and negative attitudes towards reliance on medications.** | - Conduct adherence camps for YALPH - Provide phone-based medication reminders - Connect YALPH to peer support networks. |
|  | **Mental health issues (depression, stress, trauma).**  **Chronic illnesses (Asthma, heart disease, epilepsy, allergy).** | - |
| **PHYSICAL FUNCTIONING** | Disabilities and impairments (physical, visual, hearing, learning). | - Ensure early detection, treatment and management of physical, visual, hearing, and learning disabilities. |
|  | Dissatisfaction with body image and appearance due to short stature, low body weight, prominent skin conditions, and physical disabilities. | - Counsel YALPH to boost their self-image. - The YALPH should eat nutritious food and exercise regularly to build their bodies, be physically fit and improve their body image. |
| **PSYCHOLOGICAL FUNCTIONING** | Fear of the future (unemployment due to low school attainment, finding an accepting partner to marry, HIV status disclosure to partners, financial independence). | - Support YALPH to come up with their goals for the future and to set strategies on how to achieve them. |
|  | **Grief and bereavement due to the** death of their parents and other close family members. | - |
|  | Disclosure worries and concerns due to fear of stigma and rejection. | - Provide couple counselling and support YALPH to disclose to partners. - Connect YALPH to peer support groups and camps for the exchange of knowledge and experiences on HIV status disclosure. |
| **SOCIAL FUNCTIONING** | Stressful family relationships  (conflicts, violence and abuse, stigma and discrimination, disclosure of their HIV status to other people without their consent). | - Provide family-based counseling to increase social support for YALPH. - Equip YALPH with problem-solving and conflict-resolution skills. |
|  | Limited friendships and peer relationships (due to fear of stigma and self-isolation). | - YALPH should be linked to social support networks (in-person or online, in the health facilities or community). |
|  | Challenges with forming and maintaining sexual and romantic relationships. | - Provide YALPH with comprehensive education on sexual health and relationships; encourage YALPH to bring their partners to the clinic for HIV testing, counselling and pre-exposure prophylaxis (PrEP) as needed. |
| **CHARACTERISTICS OF AN INDIVIDUAL** | Poor academic performance and attainment (due to visual and hearing impairments, learning problems, disabilities, illness, poor outlook on the future, stigma in schools). | - Early identification and intervention for visual and hearing impairments and learning problems. - Create an alternative education system that is less academic and focused on vocational or practical skills development for those who are not academically competent. |
|  | Unemployment (due to poor education attainment, lack of job skills, job scarcity, disability). | - Support YALPH in accessing opportunities for technical and vocational training, job skills development, employment and livelihood schemes. |
|  | Parenting challenges (including single parenting, financial stressors, and guilt associated with infecting their children with HIV). | - Support young parents to access post-secondary education and employment opportunities*.* - Link young parents to peer support groups for the exchange of knowledge and experiences on parenting. - Conduct male-focused sessions to teach them about fatherhood and parenting. |
|  | Transitioning out of residential care (including challenges with self-care, family conflicts, unemployment). | - YALPH should be consulted and involved in transition planning and decision-making. - After exiting from institutional care, YALPH should be assigned adult mentors to help them navigate various systems to access essential services. - Counseling should be provided to YALPH who leave institutional care on a regular basis in order to empower them to work through past traumas and current stressors. |
| **CHARACTERISTICS OF THE ENVIRONMENT** | Fear of stigma and discrimination | - Increase community education on HIV/AIDS targeting the youth. - Establish policies to assure YALPH in boarding schools and residential facilities of privacy. - Provide integrated services at healthcare facilities. |
|  | Financial stressors (due to unemployment, limited family resources, low or unreliable wages, lack of business capital, disabilities, and impairments). | Assist YALPH in gaining access to business capital schemes and job opportunities that will provide them with financial independence and alleviate financial stressors.  Teach YALPH financial management skills, including how to earn and save money. |

***Domains of the Ferrans Conceptual Model of Health Related Quality of Life. **Challenges commonly reported by participants in the in-depth interviews. ***Solutions proposed by participants.**
