# Supplementary material for: Challenges and coping strategies among young adults living with perinatally acquired HIV infection in Botswana. A qualitative study
Source: PLoS One. 2023 Apr 26;18(4):e0284467. doi: 10.1371/journal.pone.0284467 (PMC10132588; doi:10.1371/journal.pone.0284467)
Supplement: S2 File — (DOCX) [file pone.0284467.s004.docx]

**STUDY:** **CHALLENGES AND COPING STRATEGIES AMONG PERINATALLY HIV INFECTED YOUNG ADULTS IN BOTSWANA**

**INDEPTH INTERVIEW GUIDE**

**Instruction:**

Start the interview by explaining that research studies on young adults who contracted HIV from their mothers, known as perinatal transmission, have shown that having HIV has a wide range of effects on one's quality of life and well-being. This study will examine what problems young adults living with perinatal HIV (YALPH) at Botswana-Baylor face, how they cope and make recommendations on how their well-being can be improved.

1. Tell me about yourself (probe for information on age, living arrangements, education, occupation, marital status, health, and other individual characteristics).
2. Tell me about your childhood (0-12 years), adolescence (13-18 years), and now your young adulthood (18-30 years).
3. Looking at yourself now, is there something that is wrong with you? And if so, what do you think it is?
4. Tell me about times when HIV has affected your life (probe and help the person to describe their lived experiences).
5. Exactly how does HIV affect your well-being? (Probe to help the person to describe and explain their experiences).
6. What is the biggest problem that is affecting you as a young adult with HIV infection?
7. How long have you been on ART? Do you have any challenges with ART? If so, what are the challenges? What support do you need to deal with those challenges?
8. Apart from your close family members and healthcare workers, who else knows about your HIV status? If no (why?), if yes, has your relationship with this person changed since you told them about your HIV status?
9. How do you see your life in the future? Is being HIV positive a challenge?
10. Are you currently involved in a romantic relationship, or have you ever been involved in a romantic relationship? If so, have you ever had sex? Is growing up with HIV a problem? What are your needs with respect to romantic relationships and sexual activity?
11. Have you considered getting married or having children? Do you have a child? If so, what is your experience with parenting?
12. Describe your transition from adolescence to young adulthood. What helped you in the process? Looking back, what do you wish you had known, and what advice would you give to improve the transition from adolescence to young adulthood?
13. How do you feel about being HIV positive?
14. How do you cope with HIV? What helps you cope? Who do you look up to for help in coping with HIV?
15. What support do you recommend for yourself and or other young adults who contracted HIV from their mothers to promote their health and well-being? (Make reference to the issues raised throughout the interview).

**THANK YOU FOR PARTICIPATING IN THIS STUDY**
